# Determination of the Predictive Roles and Potentially Pathogenic Antigen Epitopes of α-Enolase Related to the Development of Miscarriage in Females with Autoimmune Thyroiditis

**DOI:** 10.3390/ijms24021021

**Published:** 2023-01-05

**Authors:** Jiahui Guo, Yihan Lu, Xiaoqing He, Jiashu Li, Chenling Fan, Hongmei Zhang, Weiping Teng, Zhongyan Shan, Jing Li

**Affiliations:** 1 Department of Endocrinology and Metabolism, Institute of Endocrinology, The NHC Key Laboratory of Diagnosis and Treatment of Thyroid Diseases, The First Hospital of China Medical University, Shenyang 110001, China; 2 Department of International Clinic, The Second Hospital of Dalian Medical University, Dalian 116000, China

**Keywords:** autoimmune thyroiditis, ENO1Ab, miscarriage, peptide 6, antigen epitope

## Abstract

Autoimmune thyroiditis (AIT) is a common endocrine disease which causes a significantly increased risk of miscarriage. Our recent study has shown that the increased ENO1 autoantibody (ENO1Ab) expression in an experimental AIT mouse model was induced by thyroglobulin (Tg) immunization only. In this study, we explored the potential roles of ENO1Ab in miscarriage occurrence among AIT women, and the specific epitopes of ENO1 targeted by ENO1Ab. A total of 432 euthyroid pregnant participants were selected from the project of Subclinical Hypothyroid during Early Pregnancy, including 48 women with AIT and miscarriage, 96 with miscarriage but no AIT, 96 with AIT but no miscarriage, and 192 without either AIT or miscarriage. The enzyme-linked immunosorbent assay was used to determine the serum levels of total IgG against ENO1 and 18 predicted antigen epitopes of ENO1. The results showed that women with AIT and miscarriage had the highest serum levels of ENO1Ab compared to the other groups. Logistic regression analysis showed that the serum ENO1Ab was an independent risk factor for miscarriage, especially among AIT females. The serum level of total IgG against the predicted epitope peptide 6 (i.e., P6 and aa168-183) of ENO1 was significantly increased in women with AIT and miscarriage when compared with those of both the AIT non-miscarriage group and non-AIT miscarriage group. This pilot study suggests that serum ENO1Ab may have a fair predictive value for AIT-related miscarriage, and the autoantibody specific to P6 epitope may especially be more specifically related to this disorder.

## 1. Introduction

Autoimmune thyroiditis (AIT) is one of the most prevalent autoimmune endocrine diseases, which is characterized by high serum expressions of autoantibodies against thyroglobulin (TgAb) and thyroid peroxidase (TPOAb) [1]. AIT is common among women of reproductive age and is highly associated with adverse outcomes of pregnancy [2,3,4,5]. In particular, numerous studies have shown that the risk of miscarriage in AIT pregnant females is significantly higher than that in healthy controls [3,6,7,8]. This has been attributed to thyroid dysfunction [9] and autoimmune impairment [10,11]. However, no studies have provided direct evidence for the involvement of two classical autoantibodies (e.g., TgAb and TPOAb) in the occurrence of miscarriage [12,13], or for the existence of antibody titer-dependent increase in the prevalence of miscarriage. There is also a lack of experimental evidence for their direct correlations. A previous study from our group has found that the existence of thyroid autoimmunity, represented by the positive serum TPOAb and/or TgAb, was related to an increase in the prevalence of miscarriage [3], but a higher serum thyrotrophin (TSH) level in the normal range (2.5–5.22 mIU/L) did not cause its significant alteration as compared with a lower TSH concentration (0.29–2.5 mIU/L) in pregnant women. A few studies (e.g., the postal study and tablet study) have shown that L-thyroxine supplementation could not strongly prevent miscarriage, which suggests thyroid economy deficiency cannot completely account for the increased miscarriage risk in AIT females, especially in euthyroid women [14,15]. Therefore, further explorations of the immunopathogenic mechanisms of miscarriage in females with AIT and its effective predictive markers and therapeutic targets are becoming increasingly important.

Alpha-enolase (ENO1) is a widespread and multifunctional protein, expressed in many tissues, including the thyroid and placenta tissues [16,17,18,19]. Our recent study has not only demonstrated the expression of ENO1 in thyroid tissue, but also showed significantly increased production of ENO1Ab in the Tg-immunization-induced experimental AIT mouse models [18]. Our study also found that in the mice which had received ENO1 immunization, the autoantibodies against ENO1 damaged thyroid epithelial cells, which promoted the autoimmune and inflammatory responses against thyroid tissue [18]. Meanwhile, overexpressed ENO1 protein was found in the decidua of patients who had undergone a miscarriage, and ENO1Ab could directly recognize trophoblast cells and inhibit hormone secretion [19]. Therefore, it is important to investigate whether the high expression of ENO1Ab contributes to the increased risk of miscarriage in AIT females. Moreover, epitopes are the key targets for autoimmune responses, which are also the basis to establish specific immune tolerance. In this study, we studied pregnant women to explore the association of serum ENO1Ab expression and miscarriage occurrence in euthyroid patients with AIT, and to screen for occurrences which were potentially specific to AIT-related pregnancy loss from those epitopes predicted by bioinformatics analysis, which have never been reported by the other groups before.

The findings in this pilot study could help to develop new clinical prediction and treatment strategies for AIT-related miscarriage based on ENO1Ab expression, especially those against the related specific epitopes of ENO1.

## 2. Results

### 2.1. The Correlation between Serum Antibody Expressions against ENO1Ab and the Occurrence of Miscarriage in AIT Women

The characteristics of the 432 participants from the non-AIT non-miscarriage, non-AIT miscarriage, AIT non-miscarriage, and AIT miscarriage groups were described in our previous study [20]. There were no significant differences in age, BMI, serum levels of free thyroxine (FT4), and serum ferritin (SF) between the four groups.

**Figure 1:**
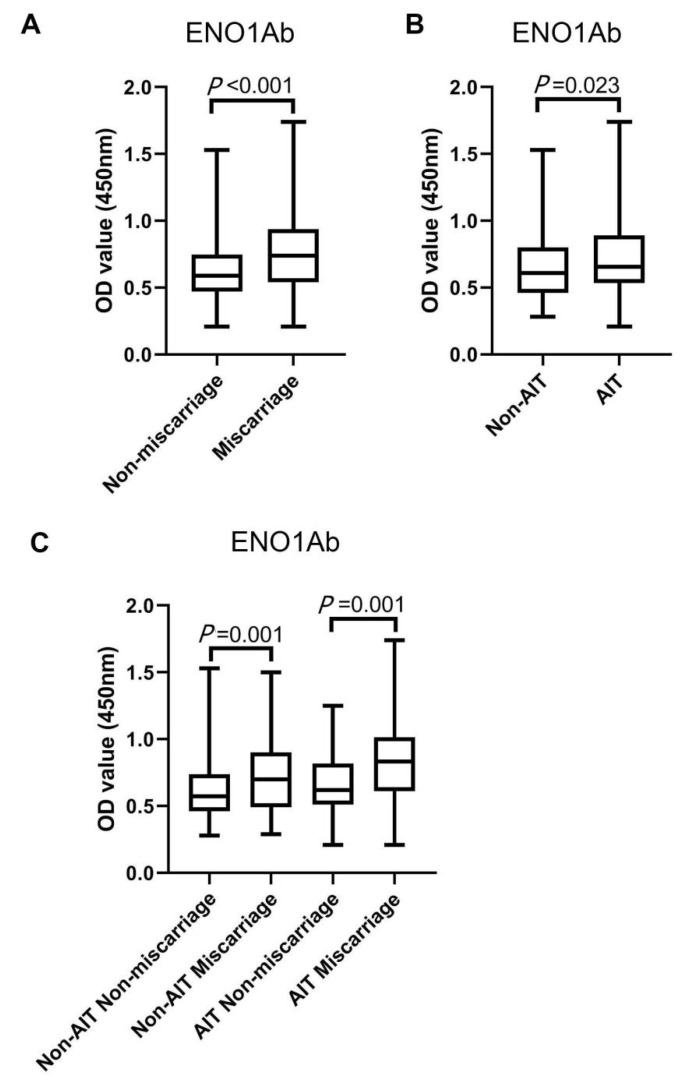
The serum levels of ENO1Ab in all euthyroid participants. The Mann–Whitney U test was performed to compare the serum levels of ENO1Ab between (**A**) participants with (*n* = 144) and those without miscarriage (*n* = 288), (**B**) participants with (*n* = 144) and and those without AIT (*n* = 288), (**C**) non-AIT participants with (*n* = 96) and those without miscarriage (*n* = 192), and AIT participants with (*n* = 48) and those without miscarriage (*n* = 96). The serum levels of total IgG against ENO1Ab were detected using enzyme-linked immunosorbent assay (ELISA) and expressed by absorbance at 450 nm. All data were exhibited as medians (P25 and P75), together with the minimum and maximum values in the box plot.

The serum total IgG against ENO1 was significantly higher in the women with miscarriage than in those without miscarriage (*p* < 0.001) (Figure 1A). It was also statistically higher in women with AIT than in those without AIT (Figure 1B). Furthermore, the serum ENO1Ab level was significantly different between AIT miscarriage and AIT non-miscarriage groups, and between non-AIT miscarriage and non-AIT non-miscarriage groups (Figure 1C).

In order to study the role of ENO1Ab in miscarriage, logistic regression analysis (LRA) was performed for those potential risk factors of miscarriage in our participants [20]. As in our previous study, participants with either a smoking or drinking history were too few to be included in the LRA [20]. According to the results of the LRA, serum ENO1Ab levels were found as an independent risk factor in both AIT (OR = 15.295) and non-AIT women (OR = 4.795) (Table 1 and Table 2). Interestingly, the serum levels of TgAb and TPOAb were not significantly correlated with an increased risk of miscarriage in euthyroid women with AIT.

**Figure 2:**
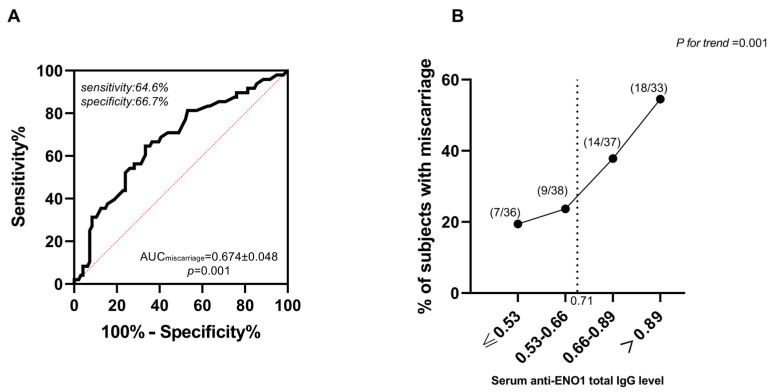
Relationship between serum levels of total IgG against ENO1 and the development of miscarriage in AIT participants. The ROC curve of the serum ENO1Ab expression for miscarriage prediction (**A**), and the titer-dependent association between serum ENO1Ab levels after quartile stratifications and the prevalence of miscarriage (**B**).

The predictive value of serum ENO1Ab levels in the occurrence of miscarriage among AIT participants was further explored by receiver operating characteristic (ROC) curves and titer-dependence trend analysis. The area under the ROC curve (AUC) of ENO1Ab was 0.674 ± 0.048 (Figure 2A). Meanwhile, according to the largest Youden index (0.313), the corresponding cut-off absorbance at 450 nm of the ENO1Ab serum level was 0.71 (Figure 2B). Every 0.1 OD value higher ENO1Ab level above the corresponding cut-off value (0.71) was related to a 39% higher risk of miscarriage in women with AIT. These results indicated that the total IgG against ENO1 may be an important independent risk factor for miscarriage in women with AIT, and the antigenic epitopes of ENO1 protein specifically contributing to the miscarriage occurrence in euthyroid women with AIT need be further explored.

The correlations between circulating ENO1Ab expression and serum FT4, TSH, TPOAb, and TgAb levels in the participants with AIT were explored by spearman correlation analysis (SCA). The results showed a negative correlation between serum ENO1Ab and TSH levels in non-miscarriage women with AIT, but not in those with pregnancy loss. There was a negative correlation between serum total IgG against ENO1 protein and TPOAb levels in AIT participants with miscarriage, but not in those without pregnancy loss (Table 3).

### 2.2. Serum Autoantibody Expressions against the 18 Predicted Antigenic Epitopes of ENO1

The serum autoantibody expressions against the predicted 18 antigenic epitopes (i.e., P1–P18) of human ENO1 protein in women randomly selected from each of the four groups (*n* = 8/group) were determined by enzyme-linked immunosorbent assay (ELISA) (Table 4). The characteristics of those 32 participants are listed in Supplementary Table S1. Interestingly, only the serum level of total IgG against P6 epitope of ENO1 (aa 168–183) was significantly increased in the AIT miscarriage group compared with that of the AIT non-miscarriage group. Autoantibodies specific to P4, P8, P9, P10, P11, P14, and P18 only showed a non-significant rise in the former group. Serum P9Ab levels were also markedly enhanced in non-AIT women with miscarriage when compared to those of the non-AIT non-miscarriage group. Autoantibodies against P1, P5, P8, P10, P15, and P18 also showed a non-significant rise in the non-AIT miscarriage group. Interestingly, the autoantibody levels against P1 and P13 were significantly decreased in women who had had a miscarriage compared with those of the non-miscarriage group among AIT subjects. In addition, the autoantibody levels against P6, P7, and P11 showed significant differences between AIT and non-AIT groups among those women who had had a miscarriage. The above findings indicated that P6 might be the specific antigenic epitope of ENO1 involved in the development of AIT-related pregnancy loss.

## 3. Discussion

Euthyroid women with AIT have been found to have a significantly higher risk for miscarriage than non-AIT ones, which cannot be decreased through L-thyroxine supplement [14]. The related pathogenic mechanisms are still unclear, and several factors potentially contribute to the occurrence of pregnancy loss [21,22]. There were some different non-organ specific autoantibodies (NOSA) found in the sera of more than 20% of women with AIT, such as autoantibodies against phospholipids, histones, and polynucleotides [23]. Those NOSA have been thought as important potential pathogenic factors for miscarriage in euthyroid women with AIT [22].

ENO1 is known as an important multifunctional protein and is distributed in many tissue cells such as thyrocytes, vascular, and trophoblast cells. It can be constructively expressed on the cell surface and in the cytoplasm and nucleus, and also maintain trafficking among the three sub-cellular locations [16,18,19,24]. ENO1Ab expression was exhibited in patients with some autoimmune and inflammatory disorders at different positive rates, such as rheumatoid arthritis (RA), systemic lupus erythematosus (SLE), antiphospholipid syndrome (APS), severe asthma, membranous nephropathy, and primary sclerosing cholangitis [25,26,27,28,29,30,31]. In fact, some of those diseases (e.g., RA, SLE, and APS) mentioned above have been reported as risk factors for spontaneous abortion [32,33,34]. Moreover, ENO1Ab has been found to be an important anti-trophoblast antibody; highly expressed in patients with unexplained recurrent miscarriages. Circulating ENO1Ab has been proposed to act as a potential biomarker for that disease [19]. It has been reported that ENO1Ab not only caused tissue cell injury through mediating antibody-dependent cytotoxicity (ADCC) and complement-dependent cytotoxicity (CDC), but also directly recognized the membrane-bound ENO1 protein and interfered with its functions [18,19,35,36]. All these findings indicate that ENO1Ab may play an important role in the pathogenesis of spontaneous abortion.

ENO1Ab expression was initially found in the sera of the patients with Hashimoto’s encephalopathy [37]. Later, we found that ENO1Ab was highly expressed in the serum of the experimental autoimmune thyroiditis (EAT) mouse model, a classical animal model of AIT [18]. Since the EAT mouse model has been established only through immunization by murine thyroglobulin and Freund adjuvant, the production of ENO1Ab may be secondary to the autoimmune responses against thyroid tissue other than the concurrent non-organ specific autoimmune disorders. Our previous study has shown that ENO1Ab caused damage to cerebral tissue in EAT mice due to a cross-autoimmune response. It has been shown that ENO1 protein is expressed in the placenta tissue as well as the thyroid gland and brain. ENO1Ab was able to directly suppress the hormone secretion function of trophoblast cells cultivated in vitro [19]. The above findings further suggest the expression of ENO1Ab may contribute to the development of spontaneous abortion in females with AIT. In the current pilot study, we systemically analyzed the relationship between serum ENO1Ab expression and the comorbidities of AIT and miscarriage. We found that the serum levels of ENO1Ab were completely different between females with and without AIT and between those with and without miscarriage. Its expression was not only significantly elevated in AIT women when compared to non-AIT women, but it was also pronouncedly higher in females suffering from miscarriage than those without a miscarriage history among either AIT or non-AIT patients. The LRA further showed that a high expression of serum ENO1Ab was an independent risk factor for miscarriage, especially in AIT females. The trend test has also directly demonstrated the titer-dependent association of pregnancy loss with ENO1Ab in AIT women and its potentially predictive value in the development of miscarriage. However, the AUC (0.674) from ROC analysis only showed a fair diagnostic value with moderate accuracy [38,39], which further reminds us of exploring the specific antigen epitope of ENO1 involved in AIT-related miscarriage.

Since serum ENO1Ab expression has been reported in a few autoimmune and inflammatory diseases, the identification of disease-specific antigen epitopes of ENO1 is gaining more and more attention to improve diagnostic and therapeutic strategies. Although multiple amino acid segments of ENO1 were found to be potentially specific epitopes to RA, cancer-associated retinopathy, lupus nephritis, and endometriosis [40,41,42,43], there is still a lack of systemic investigation into disease-specific ENO1 epitopes, and there are no reports on miscarriage. In the present study, we first predicted 18 antigenic epitopes of ENO1 from several databases by bioinformatic analysis. Then, the differential expression patterns of those epitopes were initially explored. There were significant differences in the serum levels of the autoantibodies against the predicted ENO1 epitopes between the women with and without miscarriage complicated with AIT or not. However, only serum P6Ab exhibited both significantly higher levels in AIT patients with miscarriage than those without pregnancy loss, and significantly higher levels in AIT patients when compared with those of non-AIT women who all suffered from miscarriage. Finally, the serum P6Ab levels were further detected in a total of 432 women with euthyroidism in our other study, which consisted of 48 AIT miscarriage women and matched controls in a 1:2 ratio (96 AIT patients without miscarriage, 96 non-AIT patients with miscarriage, and 192 without miscarriage) [20]. We found that serum titers of anti-ENO1-P6 total IgG and its four subtypes were not only pronouncedly increased in AIT females compared to non-AIT women, but also significantly higher in AIT females with spontaneous abortion than those without abortion. They were all independently associated with the occurrence of miscarriage in AIT patients. ROC showed that the largest AUC was 0.827 ± 0.043 for anti-ENO1-P6 IgG2 [20]. All these findings suggest that ENO1-P6Ab may be involved in the development of AIT-related miscarriage, and it can be potentially used as a good biomarker for that disorder, especially anti-ENO1-P6 IgG2. We acknowledge that we have not completed the investigation of the mechanisms of ENO1-P6Abs in the development of AIT-related miscarriage, so their cause–effect relationship awaits further study in the future.

Since ENO1 is a multiple-functioning protein, the precise activities of most of the 18 epitopes are still unclear. One previous study has suggested that the P6 epitope of ENO1 potentially contributes to the maintenance of its glycolytic enzymatic activity due to the involvement of Glu168 in the hydrogen bond formation with the PGA hydroxyl group [44]. Since placental development and energy supply in early pregnancy mainly depend on glycolysis [45], it is inferred that the direct binding of ENO1-P6 epitope and its autoantibodies may affect the glycolytic capacity of the enzyme and induce spontaneous abortion. Several studies have suggested that the IgG specific to ENO1 can directly inhibit the activities of ENO1 expressed in the cell membrane [19,36]. One in vitro study has found that membrane expression of ENO1 in MCF-7 cells, a human breast cancer cell line, was significantly increased after exposure to lipopolysaccharide (LPS), but both monoclonal and polyclonal antibodies against ENO1 were able to potentially downregulate the LPS-stimulated invasion of those cells, which was mediated by membrane ENO1 [36]. Another recent in vitro study has shown that ENO1-specific IgG could directly reduce β-hCG and progesterone production of cultured trophoblast cells, indicating a potential mechanism of causing miscarriage [19]. In addition, in vivo studies have found that ENO1Ab could damage cells through ADCC and CDC [18,35]. The constructive expression of ENO1 in the placenta [16] and increased production of P6Abs in AIT women suffering from miscarriage suggest that ENO1-P6Ab might injure the placenta and embryo through ADCC and/or CDC. We admit that these speculated pathogeneses need to be demonstrated in further experimental studies. We have established a pregnant mouse model with highly expressed ENO1-P6Ab in the serum by immunization with synthesized ENO1-P6 peptide. The fetal absorption rate, placental immune-complex deposition, energy metabolism, and other related mechanisms are being investigated. In addition, passive transfer of the IgG extracted from the above pregnant mouse model to female EAT mice without P6 peptide immunization just after mating may help decipher the role of ENO1-P6Ab in the pathogenesis of AIT-related miscarriage. At the same time, the control mice will be immunized with the synthesized mutant peptide, whose amino-acid sequence is partially changed and different from the intact ENO1-P6 epitope. In order to further investigate the exact role of the P6 epitope in the functional activities of ENO1, trophoblast cells will be cultured in vitro and their ENO1 gene will be knocked out, then they would be transfected with ENO1 gene containing either wild-type or mutated P6 coding DNA. The above experiments have been designed and will be completed in the near future.

It is still unknown why the serum levels of ENO1Abs were markedly increased in AIT patients, especially the production of ENO1-P6Ab. It has been found that more ENO1 may be expressed in the membrane of early apoptotic cells as a kind of super protein and in those under exposure to proinflammatory factors, such as tumor necrosis factor α and LPS. Previous studies have indicated that thyrocytes can express ENO1 protein [18] and many of them may develop apoptosis or be exposed to the pro-inflammatory cytokines [46,47]. We infer that the increased surface expression of ENO1 might result in the external exposure of some cryptic epitopes, and finally induce a cross-autoimmune reaction between thyroid and placenta tissues. We will also design a series of experiments to study whether the above mechanisms are existent and how the immune tolerance targeted to the ENO1-P6 epitope can be established in future work.

There are several limitations to our study. Firstly, our findings were obtained from a relatively small retrospective investigation, which awaits further confirmation in a larger-sample prospective study in the future. Secondly, the experimental studies of the mechanisms related to the production of ENO1Abs and the contribution ENO1Abs to AIT-related miscarriage have not been completed. The serum expression patterns of 18 predicted ENO1 epitopes still need to be examined in a larger-sample study using both intact and mutant epitope peptides. Thirdly, serum ENO1Ab expression has been found in some other disorders (e.g., APS, RA, and SLE), which are also suggested as risk factors for miscarriage [31,32,33,34]. Although we had excluded patients with a known personal history of other autoimmune and inflammatory disorders, we admit that we did not test those other non-organ specific autoantibodies associated with spontaneous abortion in this study, and we did not analyze the synergistic or additive actions between ENO1Ab and other NOSA, which will be performed in the future. Lastly, serum autoantibody levels show a decreasing trend from first trimester to the final trimester during normal pregnancy. According to a previous study, serum levels of antithyroid Abs were highest in the first trimester, although they were reduced by about 60% during the whole pregnancy with a half-life of 21 days [48,49]. In this study, there was a statistical difference in gestational ages between the women with and without miscarriage among the non-AIT group, the medians of which were 6 and 7 weeks of pregnancy, respectively [20]. This difference did not seem to cause a significant change in serum ENO1Ab levels between them or be the main responsible factor for the development of miscarriage in these non-AIT participants. In the future large-size investigation, gestational ages will be balanced between the different groups of patients.

In conclusion, our findings in this study have shown that ENO1Ab may be independently associated with the occurrence of miscarriage in AIT patients. A previous study has indicated that ENO1Ab expression is a novel autoimmune biomarker for unexplained recurrent pregnancy loss. This study suggests serum ENO1Ab might have a fair predictive value for AIT-related miscarriage, and especially ENO1-P6Ab may be more specifically related to this disorder. This pilot study has provided some clinical epidemiological clues for the utilization of the ENO1-P6 epitope to develop a clinical prediction biomarker of AIT-related miscarriage and establish the specific therapeutic strategy of immune tolerance. However, a large-sample clinical investigation and a series of experimental studies need to be performed to further verify our findings and to establish the cause–effect relationship between ENO1-P6Ab expression and AIT-related miscarriage.

## 4. Materials and Methods

### 4.1. Patients

All participants enrolled in this study were pregnant females in the first 4–12 weeks of pregnancy from the Subclinical Hypothyroid during Early Pregnancy (SHEP) project [20]. SHEP was a prospective cohort study performed from 2011 to 2014. The project was approved by the Ethics Committee of China Medical University ((2012)2011-32-4). Each participant was required to provide personal information, including education level, family income level, residential city, history of smoking, history of drinking, personal history of chronic diseases, personal and family history of thyroid diseases, and multiple-micronutrient supplementation. Informed consent was obtained from all patients. Fasting serum and urine samples were collected from the participants upon their first visit. Electrochemiluminescence immunoassays were used to examine TSH, FT4, TPOAb, TgAb, and SF concentrations in all participants with Cobas Elesys 601 (Roche Diagnostics, Basel, Switzerland). The ammonium persulfate method was used to determine the concentration of urinary iodine based on the Sandell–Kolthoff reaction.

A total of 9415 pregnant women from the SHEP study were recruited for our study. Participants were excluded if they had other thyroid diseases or autoimmune diseases other than AIT, if they were taking drugs affecting thyroid function, if they had hypertension, eclampsia, diabetes, a urinary iodine concentration <100 or >300 μg/L, SF < 20 μg/L, preterm labor, placenta previa, placental abruption during this pregnancy, or an induced abortion. The remaining 2054 participants were further selected based on their miscarriage history and serum TgAb and/or TPOAb levels.

**Figure 3:**
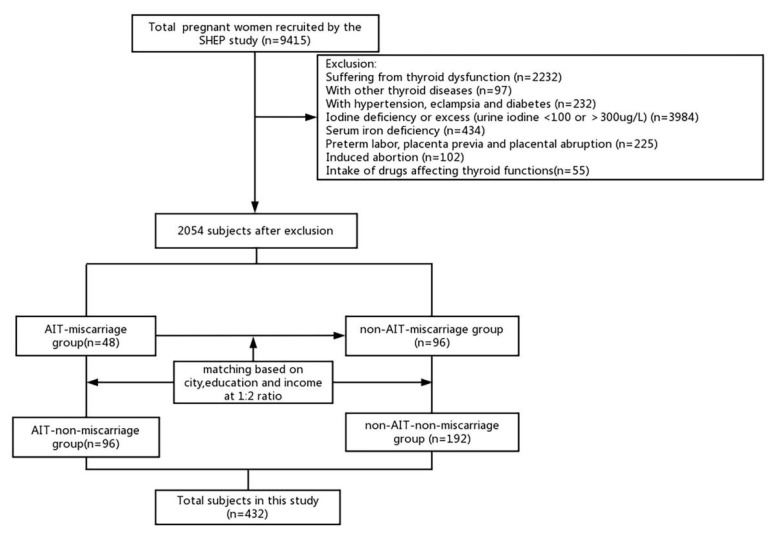
The enrollment flowchart of all the euthyroid participants in the current study.

Miscarriages that had occurred in the preceding 5 years or in the current pregnancy were investigated and counted in this study. Levels of serum TgAb > 115 IU/mL and/or TPOAb > 34 IU/L according to the manufacturer (Roche Diagnostics, Switzerland) reference range and the Guidelines on Diagnosis and Management of Thyroid Diseases during Pregnancy and Postpartum were characterized as AIT [3]. A total of 48 euthyroid miscarriage participants with AIT (AIT miscarriage group) were collected from SHEP. SPSS 23.0 was used to conduct random sampling with a ratio of 1:2 for miscarriage women without AIT (*n* = 96, non-AIT miscarriage group), AIT women without miscarriage (*n* = 96, AIT non-miscarriage group), and those without either AIT or miscarriage (*n* = 192, non-AIT non-miscarriage group). The matching factors were city of residence, education level, and family income level. Finally, a total of 432 participants were included in this study (Figure 3).

### 4.2. Prediction of the Potential Epitopes in ENO1 Protein

**Figure 4:**
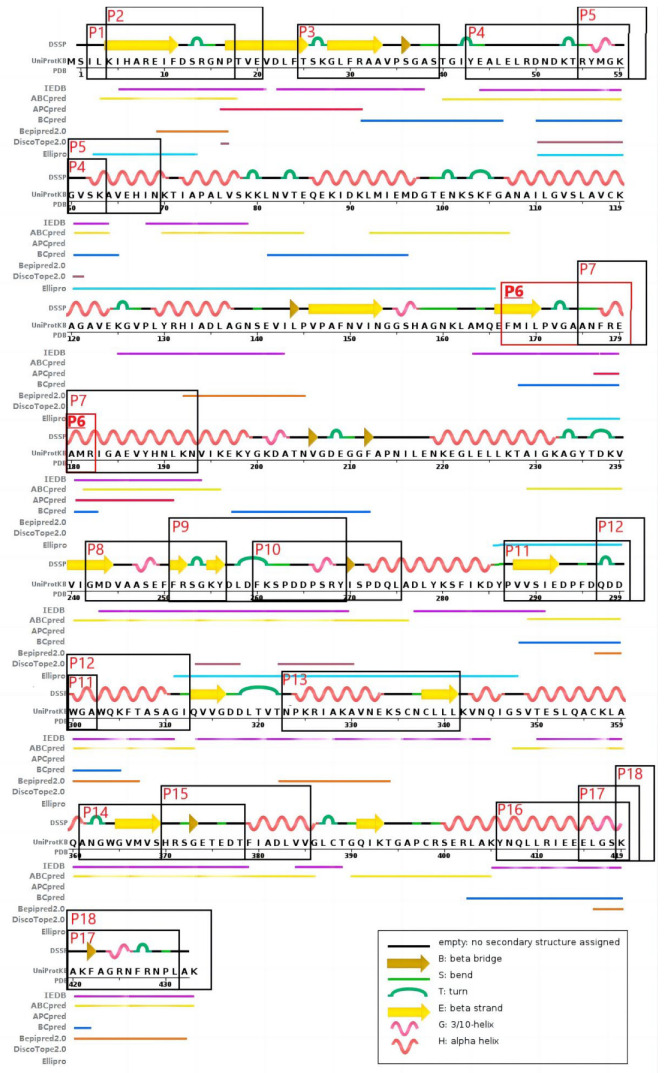
The 18 selected antigenic epitopes of human ENO1 protein predicted by databases.

The amino acid sequence (PubMed protein database, GI: 1167843), secondary structure, and 3D structure (PDB database, PDB ID: 2PSN) of the human ENO1 protein were used to predict its potential antigenic epitopes by bioinformatics [50]. Antigenic epitopes recognized by B cells were predicted by the following network servers according to the amino acid sequence: ABCpred, APCpred, Bcpred, Bepipred 2.0, and IEDB. Antigenic epitopes based on crystal structure were predicted by the DissoTope2.0 and Ellipro web servers. The epitopes recognized by B cells which could be predicted by at least two web servers were selected for further study. Antigenic epitopes recognized by T cells were predicted by the IEDB database. A total of 18 antigenic epitopes in the human ENO1 protein were further explored in this study, named P1 to P18 according to their location on the protein, and synthesized and purified by Sangon Biotech (Shanghai, China) (Table 5, Figure 4).

### 4.3. Enzyme-Linked Immunosorbent Assay

The serum levels of autoantibodies against the ENO1 protein and its 18 related epitopes of ENO1 were determined by ELISA as previously described [52]. Microtiter plates coated with recombinant ENO1 (1 μg/well) or the 18 recombinant peptides (10 ng/well) were incubated at 4 °C overnight and blocked with 1% bovine serum albumin (Sigma-Aldrich, St. Louis, MO, USA). Serum levels of ENO1Ab were measured at a dilution of 1:50, and serum levels of autoantibodies against the 18 epitopes were measured at a dilution of 1:250 in participants from our study. HRP-labeled rabbit anti-human total IgG antibodies (Bioss, Beijing, China) were used as secondary antibodies at a dilution of 1:1000. The enzymatic reaction was measured by TMB, and color development was stopped using hydrochloric acid at room temperature. Absorbance was measured at 450 nm using Infinite 2000 PRO (TECAN, Port Melbourne, VIC, Australia).

### 4.4. Statistical Analysis

The data were analyzed using SPSS 23.0 statistical software. The skewed distributed variables were reported as the medians with 25–75th percentiles for continuous variables. The normally distributed variables were reported as the means ± SD. The Mann–Whitney U test with a skewed distribution and a two-sample *t*-test were used to analyze variables with a normal distribution. Categorical variable frequencies were compared by the chi-squared test. SCA was performed to examine the relationship between ENO1Ab and serum levels of FT4, TSH, TPOAb, and TgAb. The independent risk variables for miscarriage were evaluated by LRA. ROC curve analysis was used to determine the optimal cut-off values of ENO1Ab in participants with AIT and miscarriage. The titer-dependent association between serum ENO1Ab levels and miscarriage prevalence in participants with AIT was examined by the trend test. Differences were considered statistically significant if *p* < 0.05 in two groups or *p* < 0.017 (0.05/3) or 0.0125 (0.05/4) for adjusted *p* values in multiple comparisons.

## Figures and Tables

**Table 1:** Logistic regression of potential risk factors of miscarriage in euthyroid pregnant women with AIT.

Variants	B	SE	Wald	*p*	OR (95%CI)
Age (years)	0.085	0.063	1.799	0.180	1.088 (0.962–1.231)
Gestational age (weeks)	−0.166	0.161	1.062	0.303	0.847 (0.618–1.161)
BMI (kg/m^2^)	−0.027	0.078	0.115	0.735	0.974 (0.835–1.136)
Urine iodine/creatinine (μg/g)	0.007	0.005	1.985	0.159	1.007 (0.997–1.017)
Serum ferritin (μg/L)	0.004	0.004	0.96	0.327	1.004 (0.996–1.012)
TSH (mIU/L)	0.403	0.254	2.514	0.113	1.497 (0.909–2.464)
FT4 (pmol/L)	−0.133	0.151	0.773	0.379	0.875 (0.651–1.178)
TPOAb (IU/mL)	0.001	0.002	0.301	0.583	1.001 (0.998–1.004)
TgAb (IU/mL)	−0.001	0.001	1.106	0.293	0.999 (0.997–1.001)
ENO1Ab ^a^	2.728	0.921	8.773	0.003	15.295 (2.516–92.982)

^a^ Serum levels of ENO1Ab were detected using ELISA and reflected by absorbance at 450 nm.

**Table 2:** Logistic regression of potential risk factors of miscarriage in euthyroid pregnant women without AIT.

Variants	B	SE	Wald	*p*	OR(95%CI)
Age (years)	0.095	0.04	5.642	0.018	1.099 (1.017–1.189)
Gestational age (weeks)	−0.695	0.138	25.519	<0.001	0.499 (0.381–0.654)
BMI (kg/m^2^)	−0.04	0.038	1.113	0.291	0.96 (0.891–1.035)
Urine iodine/creatinine (μg/g)	−0.002	0.002	0.991	0.32	0.998 (0.995–1.002)
Serum ferritin (μg/L)	0.002	0.003	0.479	0.489	1.002 (0.997–1.007)
TSH (mIU/L)	0.173	0.165	1.103	0.294	1.189 (0.86–1.644)
FT4 (pmol/L)	−0.006	0.087	0.006	0.941	0.994 (0.838–1.178)
ENO1Ab ^a^	1.568	0.56	7.834	0.005	4.795 (1.6–14.37)

^a^ Serum levels of ENO1Ab were detected using ELISA and reflected by absorbance at 450 nm.

**Table 3:** Spearman correlation analysis of ENO1Ab expression with serum levels of FT4, TSH, TPOAb, and TgAb in AIT women.

ENO1Ab	FT4	TSH	TPOAb	TgAb
AIT non-miscarriage group	rs = 0.078	rs = −0.201	rs = 0.131	rs = −0.039
(*n* = 96)	NS	0.049	NS	NS
AIT miscarriage group	rs = −0.009	rs = −0.055	rs = −0.311	rs = −0.171
(*n* = 48)	NS	NS	0.031	NS

NS, non-significant.

**Table 4:** The serum levels of autoantibodies against P1–P18 antigen epitopes of ENO1.

	Group				*p* Value		
	Non-AIT		AIT		Non-AIT	AIT	Miscarriage
	Non-Miscarriage (*n* = 8)	Miscarriage (*n* = 8)	Non-Miscarriage (*n* = 8)	Miscarriage (*n* = 8)	Miscarriage vs. Non-Miscarriage	Miscarriage vs. Non-Miscarriage	AIT vs. non-AIT
P1Ab ^a^	0.37 (0.16–0.93)	0.94 (0.37–1.20)	0.71 (0.38–2.00)	0.16 (0.13–0.47)	0.189	0.016 ^b^	0.018
P2Ab ^a^	0.67 (0.57–0.88)	0.40 (0.32–0.80)	0.48 (0.27–0.60)	0.31 (0.23–0.50)	0.093	0.248	0.248
P3Ab ^a^	0.48 (0.42–0.72)	0.29 (0.20–0.57)	0.69 (0.45–0.97)	0.60 (0.34–0.87)	0.141	0.563	0.115
P4Ab ^a^	1.07 (0.77–1.45)	0.22 (0.17–0.68)	0.55 (0.25–1.00)	0.84 (0.47–1.03)	0.046	0.401	0.036
P5Ab ^a^	0.17 (0.13–0.36)	0.45 (0.24–1.00)	0.34 (0.28–0.69)	0.31 (0.12–1.09)	0.040	0.752	0.344
P6Ab ^a^	0.29 (0.15–0.46)	0.23 (0.16–0.71)	0.46 (0.41–0.59)	1.05 (0.86–1.14)	0.752	0.005 ^b^	0.013 ^b^
P7Ab ^a^	1.19 (0.65–1.38)	0.71 (0.59–1.10)	0.37 (0.28–0.48)	0.24 (0.17–0.40)	0.207	0.103	0.005 ^b^
P8Ab ^a^	0.62 (0.26–0.90)	0.70 (0.39–1.10)	0.76 (0.43–1.14)	0.83 (0.70–0.89)	0.462	0.916	0.529
P9Ab ^a^	0.71 (0.46–0.87)	1.04 (0.88–1.21)	0.73 (0.30–1.10)	1.13 (0.49–1.18)	0.013 ^b^	0.247	1.000
P10Ab ^a^	0.52 (0.15–1.06)	1.02 (0.63–1.28)	0.23 (0.13–0.88)	0.59 (0.18–0.79)	0.156	0.431	0.052
P11Ab ^a^	0.23 (0.17–0.26)	0.15 (0.12–0.20)	0.27 (0.23–0.45)	0.30 (0.23–0.42)	0.03	0.958	0.002 ^b^
P12Ab ^a^	0.79 (0.53–1.67)	0.43 (0.23–0.64)	0.55 (0.48–1.29)	0.35 (0.12–0.58)	0.036	0.052	0.400
P13Ab ^a^	1.00 (0.40–1.22)	0.26 (0.23–0.78)	0.74 (0.44–1.10)	0.20 (0.15–0.39)	0.046	0.003 ^b^	0.083
P14Ab ^a^	0.65 (0.58–0.78)	0.56 (0.23–0.90)	0.51 (0.44–0.66)	0.68 (0.47–0.85)	0.431	0.293	0.462
P15Ab ^a^	0.83 (0.34–1.27)	0.93 (0.45–1.51)	0.90 (0.34–1.16)	0.76 (0.61–1.09)	0.461	0.958	0.401
P16Ab ^a^	1.52 (1.29–1.85)	1.14 (0.98–1.46)	1.01 (0.84–1.16)	0.80 (0.36–1.07)	0.115	0.115	0.021
P17Ab ^a^	0.80 (0.56–0.81)	0.53 (0.32–0.76)	0.51 (0.42–0.60)	0.37 (0.23–0.62)	0.045	0.208	0.270
P18Ab ^a^	0.20 (0.17–0.24)	0.29 (0.23–0.32)	0.20 (0.18–0.40)	0.22 (0.17–0.30)	0.114	0.526	0.343

^a^ Serum levels of total IgG against P1–P18 antigen epitopes were reflected by absorbance at 450 nm. ^b^ *p* value for multiple comparisons was 0.017 as the cutoff value indicating statistical significance.

**Table 5:** The 18 selected antigenic epitopes of human ENO1 protein predicted by databases.

No.	Amino Acid Sequence	Location of Epitope	Length of Peptide	Database	Reported Potential Function
P1	ILKIHAREIFDSRGNP	3–18	16	IEDB, ABCpred, Ellipro	NA
P2	KIHAREIFDSRGNPTVE	5–21	17	IEDB, Bepipred2.0, DiscoTope2.0	NA
P3	TSKGLFRAAVPSGAS	26–40	15	IEDB	Related to rheumatoid arthritis [43]
P4	YEALELRDNDKTRYMGKGVSK	44–64	21	IEDB, ABCpred, DiscoTope2.0, Ellipro	Related to cancer-associated retinopathy syndrome and endometriosis [40,41]
P5	RYMGKGVSKAVEHIN	56–70	15	IEDB	Related to cancer-associated retinopathy syndrome and endometriosis [40,41]
P6	FMILPVGAANFREAMR	168–183	16	IEDB, BCpred, Ellipro	A functional glycolytic segment [44]
P7	ANFREAMRIGAEVYHNLKN	176–194	19	IEDB, ABCpred, APCpred	NA
P8	GMDVAASEFFRSGKY	243–263	21	IEDB, ABCpred	A functional glycolytic segment [44]
P9	FRSGKYDLDFKSPDDPSRY	252–270	19	IEDB, ABCpred, DiscoTope2.0, Ellipro	A functional glycolytic segment anda plasminogen-binding site [44,51]
P10	FKSPDDPSRYISPDQL	261–276	16	ABCpred, DiscoTope2.0	A functional glycolytic segment [44]
P11	PVVSIEDPFDQDDWGA	288–307	16	ABCpred, BCpred, Bepipred2.0	
P12	QDDWGAWQKFTASAGI	298–313	16	IEDB, ABCpred	NA
P13	NPKRIAKAVNEKSCNCLL	324–341	18	IEDB, Bepipred2.0	NA
P14	ANGWGVMVSHRSGETEDT	362–379	18	IEDB, ABCpred	NA
P15	HRSGETEDTFIADLVV	371–389	19	IEDB, ABCpred	
P16	YNQLLRIEEELGSKAK	405–422	16	IEDB, BCpred	Binding with c-myc promoter [44]
P17	ELGSKAKFAGRNFRNPL	415–432	18	IEDB, Bepipred2.0	Binding with c-myc promoter [44]
P18	KAKFAGRNFRNPLAK	420–434	15	IEDB	Binding with c-myc promoter [44]

## Data Availability

The article contains all of the findings obtained from the study.

## Supplementary Materials

The following supporting information can be downloaded at: https://www.mdpi.com/article/10.3390/ijms24021021/s1.
