# Supplementary material for: Determination of the Predictive Roles and Potentially Pathogenic Antigen Epitopes of α-Enolase Related to the Development of Miscarriage in Females with Autoimmune Thyroiditis
Source: Int J Mol Sci. 2023 Jan 5;24(2):1021. doi: 10.3390/ijms24021021 (PMC9862122; doi:10.3390/ijms24021021)
Supplement: Supplementary file 1 [file ijms-24-01021-s001.zip › ijms-2034876-supplementary.pdf]

Table S1. Characteristics of representative participants in the study of serum P1-P18 antigenic epitopes antibodies.

	Group				*P* value			
	Non-AIT		AIT		Non-AIT	AIT	Non-miscarriage	Miscarriage
	Non-miscarriage (*n*=8)	Miscarriage (*n*=8)	Non-miscarriage (*n*=8)	Miscarriage (*n*=8)	Miscarriage vs. non-miscarriage	Miscarriage vs. non-miscarriage	AIT vs. non-AIT	AIT vs. non-AIT
Age, years	27.5±3.3	32.3±4.6	26.4±4.2	30.5±4.0	NS	NS	NS	NS
Gestational age, weeks	5.5(5.0-7.0)	5.0(4.3-7.0)	6.0(5.3-7.0)	5.5(4.3-6.0)	NS	NS	NS	NS
BMI, Kg/m^2	21.7(19.2-22.6)	23.5(22.0-25.5)	24.0(21.5-24.8)	21.2(19.4-26.9)	NS	NS	NS	NS
Smoking (%)	1 (12.5)	1 (12.5)	0 (0)	0 (0)	NS	NS	NS	NS
Drinking (%)	1 (12.5)	0 (0)	0 (0)	0 (0)	NS	NS	NS	NS
TSH, mIU/L	2.06(1.07-2.38)	1.6(1.48-2.29)	2.34(1.55-2.97)	2.59(2.16-3.63)	NS	NS	NS	NS
FT4, pmol/L	16.78±2.28	15.66±1.40	16.50±1.66	15.56±1.30	NS	NS	NS	NS
TPOAb, IU/ml	5.46(5.00-13.60)	8.47(6.18-11.89)	321.35(152.58-536)	231.05(180.23-367.9)	NS	NS	0.001	0.001
TgAb, IU/ml	38.41(11.53-76.91)	14.19(12.25-20.06)	327.05(276.85-429.53)	272.30(205.10-404.95)	NS	NS	0.001	0.001
Serum ferritin, μg/L	44.72(36.91-111.46)	65.37(39.84-114.05)	71.46(51.19-115.53)	62.865(53.66-75.80)	NS	NS	NS	NS
Urinary iodine/creatinine, μg/g	104.63(59.72-131.76)	127.78(102.14-155.12)	79.73(63.42-102.36)	93.47(72.22-145.01)	NS	NS	NS	NS

The skewed distributed variables are reported as the median (25-75th percentiles) for continuous variables. The above data are presented as n (%) for categorical variables or median (25-75th percentiles) for continuous variables. *P* value less than (0.05/4 = 0.0125) is considered statistically significant after corrected for multiple comparisons using chi-square test.

The Mann-Whitney U test was used to analyze variables with a skewed distribution. Differences were considered statistically significant if $P < 0.05$ for two groups or $P <$ (0.05/3=0.017 or

0.05/4=0.0125) for adjusted *P* value for multiple comparisons.

NS, non-significant.
